# Spättoxizität nach adjuvanter Strahlentherapie von Zervixkarzinomen – Vergleich von konventionellen Techniken mit der „image-guided intensity-modulated radiotherapy“

**DOI:** 10.1007/s00066-022-01935-0

**Published:** 2022-04-25

**Authors:** G. G. Grabenbauer, Artem Trofymov

**Affiliations:** 1 Radioonkologie und Strahlentherapie, Onkologisches Zentrum Klinikum Coburg, Coburg, Deutschland; 2 Universitätsklinikum Erlangen, Erlangen, Deutschland

## Hintergrund

Zielsetzung der Studie war es, zu prüfen, ob die *gastrointestinale Spätmorbidität* des Grades 2 und mehr nach der postoperativen Radiochemotherapie (RCT) bei Patientinnen mit einem Zervixkarzinom verringert werden kann, und zwar im randomisierten Vergleich zwischen einer intensitätsmodulierten Radiotherapie (IG-IMRT) und einer 3d-konformalen Radiotherapie (3dCRT).

## Patientinnen und Methoden

Von 2011 bis 2019 wurden insgesamt 300 Patientinnen randomisiert. Zwei der folgenden drei Risikofaktoren mussten für den Studieneinschluss erfüllt sein: Tumorgröße > 4 cm, tiefe Stromainvasion (> 50 %), Lymphangiosis carcinomatosa. Die verschriebene Dosis betrug 25 × 2 Gy, wobei 95 % des Planungszielvolumens (PTV) 95 % der Gesamtdosis erhalten sollten. Die 3dCRT erfolgte mit einer 4‑Felder-Box-Technik und einmal wöchentlicher Kontrollaufnahme. Die IG-IMRT mit täglicher Verifikationskontrolle hatte als Randbedingungen für die Dünndarmbelastung V15 Gy < 190 ml, V40 Gy < 100 ml. Alle Patientinnen erhielten einen Brachytherapie-Boost in die Scheide (2 × 6 Gy/5 mm Tiefe). Patientinnen, die *einen *der folgenden „High-risk“-Faktoren (Infiltration der Parametrien, pN+, R1) aufwiesen, wurden mit einer 1‑mal wöchentlichen, simultanen Chemotherapie unter Verwendung von Cisplatin (40 mg/m^2^) behandelt. Primärer Endpunkt der Studie war die kumulative Inzidenz einer gastrointestinalen Spättoxizität von Grad 2 und mehr (CTC v3.0) nach drei Jahren. Sekundäre Endpunkte waren die akute Toxizität, die Lebensqualität und die Überlebensraten sowie die Evaluation des Rezidivmusters.

## Ergebnisse

Nach einer medianen Nachbeobachtungszeit von 46 Monaten (IQR: 20–72 Mon.) betrug die kumulative 3‑Jahres-Inzidenz einer gastrointestinalen Nebenwirkung von Grad 2 und mehr nach IG-IMRT 21 % und nach 3dCRT 42 % (HR 0,46; 95 %-CI 0,29–0,73, *p* < 0,001). Insbesondere Symptome, wie die chronische Diarrhö (*p* = 0,04), Appetit (*p* = 0,008) und Darmbeschwerden (*p* = 0,002) wurden durch die IG-IMRT positiv beeinflusst. Weder die lokoregionär rezidivfreie (82 % vs. 84 %, n. s.) noch die krankheitsfreie Überlebensrate (77 % vs. 81 %, n. s.) wurde von den unterschiedlichen Bestrahlungstechniken (IG-IMRT vs. 3dCRT) beeinflusst.

## Schlussfolgerung der Autoren

Die IG-IMRT halbiert im Vergleich zur 3dCRT die Rate an relevanten gastrointestinalen Spättoxizitäten bei der postoperativen RCT des Zervixkarzinoms.

## Kommentar

Zunächst darf der Erstautorin und Studienleiterin Dr. Supriya Chopra vom Tata Memorial Hospital in Mumbai zu diesen exzellenten Resultaten gratuliert werden. Sie leistete damit einen wesentlichen Beitrag zur Verbesserung der Behandlungsresultate und insbesondere der doch nicht unerheblichen Langzeitfolgen einer kombinierten operativen und radioonkologischen Behandlung von Patientinnen mit einem Zervixkarzinom. Bis dato gab es in diesem Kontext lediglich Daten zur gastrointestinalen Spättoxizität (von Grad 2–4), die nicht über einen Nachbeobachtungszeitraum von 12 bis 24 Monaten hinausgingen. Nach alleiniger postoperativer RT wurden diese im Rahmen der PORTEC-3-Studie [1] mit knapp 40 %, nach RCT sogar über 40 % (mit einem sehr geringen Anteil an Patientinnen mit einer Grad-4-Toxizität) in einem erschreckend hohen Prozentsatz berichtet. Allerdings wurde dabei nicht genau spezifiziert, mit welcher Technik bestrahlt wurde.

Ist die Fragestellung IMRT vs. 3dCRT überhaupt noch zeitgemäß? Bestrahlen wir nicht ausnahmslos pelvine Zielvolumina mit den eleganten VMAT-Techniken, und das schon seit geraumer Zeit? So berichten Viani et al. [3] bereits 2016 mit guter Evidenz im Rahmen einer randomisierten Studie zum Prostatakarzinom, dass die maximale gastrointestinale Spättoxizität von Grad ≥ 2 lediglich 6,4 % nach der IMRT im Vergleich zu 21,7 % (*p* < 0,001) nach 3dCRT betrug. Aber bitte, legen wir die arrogante „westliche Brille“ zur Seite und vergegenwärtigen uns, dass – global gesehen – ca. 85 % aller Zervixkarzinompatientinnen nicht hier, sondern in sich entwickelnden Ländern mit deutlich geringeren technischen Ressourcen behandelt werden müssen [4].

Interessant erscheint uns noch der Blick auf das Rezidivmuster im Rahmen der hier zu diskutierenden Studie. Erwartungsgemäß führend war die Rate an Fernmetastasen bei 45 von 300 (15 %) der Patientinnen. Details zur Lokalisation der pelvinen und paraaortalen Rezidive wurden inzwischen im *„Red Journal“* zur Publikation angenommen [2]. Die Rate an pelvinen Rezidiven war mit 24/300 (8 %) niedrig, die der paraaortalen Rezidive mit 9/300 (3 %) noch niedriger. Interessant in diesem Zusammenhang ist, dass die Region des Scheidenabschlusses überproportional häufig betroffen war, was Anlass zur Modifikation der Zielvolumendefinition und ggf. der operativen Therapie geben sollte.

### Fazit

Zusammenfassend bleibt festzustellen, dass nach der Operation die IG-IMRT mit 25 × 2 Gy und simultaner Cisplatinchemotherapie die Standardbehandlung von Patientinnen mit einem „High-risk“-Zervixkarzinom sein sollte.

*Gerhard G. Grabenbauer und*

*Artem Trofymov, Ph.D. (Univ. Charkiw, Ukraine)*
